# Language-related domain-specific and domain-general systems in the human brain

**DOI:** 10.1016/j.cobeha.2018.04.008

**Published:** 2018-06

**Authors:** Karen L Campbell, Lorraine K Tyler

**Affiliations:** 1 Department of Psychology, Brock University, St. Catharines, ON L2S 3A1, Canada; 2 Department of Psychology, University of Cambridge, Cambridge CB2 3EB, United Kingdom

## Abstract

While a long history of neuropsychological research places language function within a primarily left-lateralized frontotemporal system, recent neuroimaging work has extended this language network to include a number of regions traditionally thought of as ‘domain-general’. These include dorsal frontal, parietal, and medial temporal lobe regions known to underpin cognitive functions such as attention and memory. In this paper, we argue that these domain-general systems are not required for language processing and are instead an artefact of the tasks typically used to study language. Recent work from our lab shows that when syntactic processing — arguably the only domain-specific language function — is measured in a task-free, naturalistic manner, only the left-lateralized frontotemporal syntax system and auditory network are activated. When syntax is measured within the context of a task, several other domain-general networks come online and are functionally connected to the frontotemporal system. While we have long argued that syntactic processing does not occur in isolation but is processed in parallel with semantics and pragmatics — functions of the wider language system — our recent work makes a strong case for the domain-specificity of the frontotemporal syntax system and its autonomy from domain-general networks.

**Current Opinion in Behavioral Sciences** 2018, **21**:132–137

This review comes from a themed issue on **The evolution of language**

Edited by **Christopher Petkov** and **William Marslen-Wilson**

For a complete overview see the Issue and the Editorial

Available online 1st June 2018

**https://doi.org.10.1016/j.cobeha.2018.04.008**

2352-1546/© 2018 The Authors. Published by Elsevier Ltd. This is an open access article under the CC BY license (http://creativecommons.org/licenses/by/4.0/).

While a long history of neuropsychological research on the neural substrate of language has argued for a primarily left hemisphere language system [1, 2, 3•], more recent neuroimaging research has drawn attention to the wider neural context for language function. This wider context sees the neural basis for language in terms of a coalition of domain-specific and domain-general neural systems [4]. The basic concept underpinning this dichotomy is that some neural networks or regions are specialized to carry out language-specific functions such as syntax (e.g. [5]), whereas others are domain-general serving cognitive functions such as attention and memory, which apply across a variety of cognitive domains. These domain-general regions coactivate with language-specific regions when language functions, such as syntactic processing, become difficult due to temporary ambiguities or the presence of discontinuous dependencies [6, 7]. However, it remains unclear how these domain-general regions contribute to language processing and, indeed, whether they are even necessary for the processing of natural, everyday language. In our view, syntactic processing is the only plausible domain-specific aspect of language processing, and the syntax system can be differentiated both from the wider language system (including domain-general components responsible for semantics and pragmatics) and from broader domain-general networks. In this paper, we present a novel, data-driven approach for isolating the domain-specific syntax system and argue that natural syntactic processing does not require domain-general input, even when it is ‘difficult’ due to, for instance, temporary syntactic ambiguity.

## Tasks activate domain-general networks

In our view, previous attempts to delineate domain-specific from domain-general systems have suffered from three critical flaws. First, this work has failed to isolate domain-specific language functions (in our view, syntax; see also ([8], this volume)) from more general language processes that are also called upon by other domains (such as semantics and pragmatics). Second, this work has almost invariably tested language comprehension within the context of a task, which places a variety of cognitive demands on participants apart from language comprehension. Domain-general regions may be active during these tasks because they are contributing to language processing or, more likely, because they are contributing to general attention and memory demands of the task. Third, previous neuroimaging work has primarily relied on univariate analysis techniques, which can only show that different regions are active at the same time, but provides no information on how these regions covary together or separate into functionally differentiable systems (cf. [9]).

**Figure 1:**
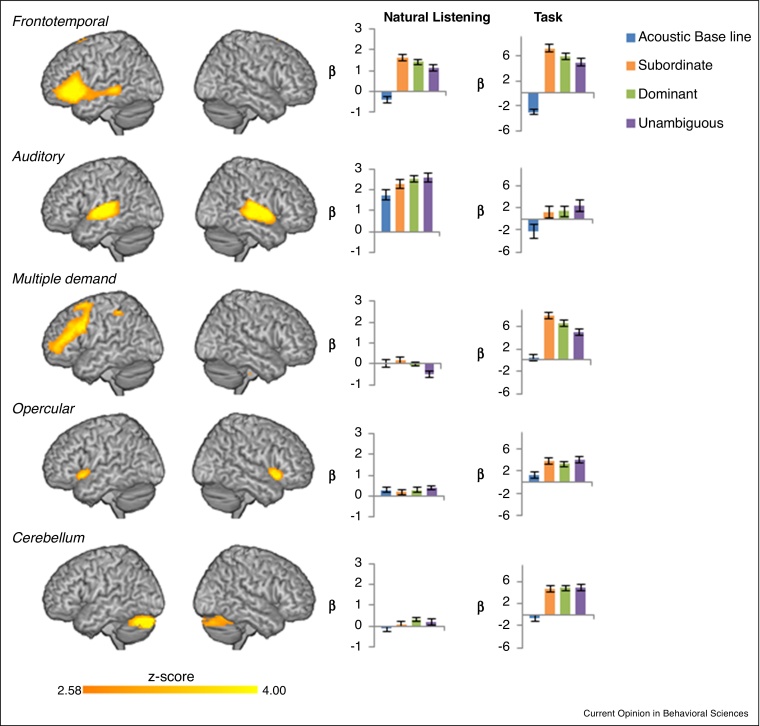
Independent components analysis of young adult data alone (*N* = 35, age range: 22–45 years; subset of data from Campbell *et al.* [2016]). Left panel shows the group average spatial map for each component rendered on a canonical brain. Right panel shows mean loading values for each network during Natural Listening and Task for the four conditions (acoustic baseline, subordinate, dominant, and unambiguous). Error bars = standard errors.

We suggest that a better approach to separating domain-specific from domain-general systems is to use a data-driven method, such as independent components analysis (ICA; [10, 11]), which decomposes the ongoing fMRI signal into a set of independent components or sets of regions that covary together over time. We recently used this approach to differentiate between domain-general networks and the domain-specific syntax system, and found that domain-general networks are only implicated during task-based language comprehension [12•, 13]. In one study, we found that when syntactic processing is measured within the context of natural listening (for example, when participants listen to sentences containing a temporary syntactic ambiguity such as: ‘*bullying teenagers*’ in the sentence ‘*The newspaper reported that **bullying teenagers** are a problem for the local school*’) and do not have an additional task to perform, only the left-lateralized frontotemporal syntax system (e.g. [3•, 14, 15]) and auditory networks are activated, and these are functionally connected to each other. In contrast, if syntactic processing is measured within the context of even a simple task (i.e. participants listen to the same sentences containing syntactic ambiguities and judge the acceptability of a single continuation verb following the syntactically ambiguous phrase), then apart from the left frontotemporal and auditory networks several domain-general networks are also activated (including the multiple demand [16] and salience/opercular network [17]). Analyses of the functional connectivity between these networks show that the left hemisphere frontotemporal syntax network is only connected to these domain-general networks during overt task performance, and not during natural listening, further emphasizing the sufficiency of the left frontotemporal network for syntactic analyses during natural language comprehension. While this study used a lifespan sample, ranging in age from 18 to 88 years, there was no age effect, suggesting that this pattern does not change during adulthood. Moreover, we have replicated these results in younger adults alone (see Figure 1), suggesting that it is not simply an artefact of our ageing sample. Thus, the manner in which language functions are tested is critical, and previous reports of domain-general involvement in language comprehension [6, 7] may likely reflect the impact of general task demands.

Data-driven methods, such as ICA, are only useful if the components (or networks) they identify track with the nature of the input. That is, while most resting state ICA papers would lead us to believe that there are a set of canonical networks and the structure of these networks is largely unchanging from state to state [18, 19, 20], this is not the case. Patterns of functional connectivity dynamically shift to meet changing situational demands [21, 22, 23]. Regions that are not connected in one context may become connected in another context to jointly carry out a particular function, and it is critical that ICA be able to detect these shifts in ‘process specific alliances’ [24].

In our experience, ICA is sensitive to shifting cognitive demands, as observed when we used ICA to delineate functional networks active during naturalistic viewing — that is, as participants watched a movie [25^•^]. In contrast to the left-lateralized frontotemporal syntax network that was related to syntactic processing in our earlier ICA study [12^•^], an ICA of the movie data identified the wider language network consisting of a bilateral frontotemporal system that correlated strongly with the language spoken during the movie (TRs were coded as 1's or 0's depending on whether or not someone in the film was speaking, and this language timecourse was convolved with the canonical HRF and then correlated with the network timecourses). This wider language network was independent from, but functionally connected to, a bilateral auditory network and regions of the dorsal default network [26, 27]. Given that the language spoken in the movie is accompanied by visual input and is phonologically, syntactically, semantically and pragmatically rich, it is unsurprising that we now see the language network extending beyond the left-lateralized syntax system. Clearly, ICA is sensitive enough to detect shifting network structure depending on the nature of the input. Thus, if one wants to identify the system which underpins a function that is specific to the language domain — that is syntactic processing — then input to the system should focus on those aspects of language that drive this sort of processing.

## Defining domain-specificity for language

The important point here is that for the distinction between domain-specific and domain-general systems to be explanatory, the ‘domain-specific’ neural system for language needs to be defined. This has not been the approach used in most studies attempting to contrast domain-specific with domain-general processes in language. For instance, Fedorenko and colleagues [4, 9, 28, 29, 30•] typically use an operational definition of the domain-specific language system as the brain regions involved in reading sentences after accounting for regions involved in reading sequences of pronounceable nonwords (presented one word/nonword at a time for ∼350 ms each, followed by a memory probe testing recognition of one of the previously viewed words/nonwords). Further analyses are restricted to regions identified by this ‘localizer’. Putting aside the fact that reading may call upon different systems than spoken language,^3^ we have already demonstrated that tasks, even simple ones such as this one, lead to the activation of additional domain-general regions [12•, 13]. Thus, it is unsurprising that by this method, Fedorenko's definition of the domain-specific language system involves both an extensive left hemisphere fronto-temporal-parietal system and frontal and parietal regions in the right hemisphere [6]. Defined in this way, the domain-specific ‘language network’ includes a broad set of language functions including lexical, syntactic, semantic and pragmatic functions. However, not all of these components would be considered to be equally domain-specific to language. Few would argue for the domain-specificity of semantics or pragmatics since they are involved in many cognitive functions which do not involve language. Moreover, the neural regions involved in semantics during language processing partially overlap with those involved in other cognitive activities, such as object recognition [31^•^]. Nevertheless, these language-related domain-general systems are an integral part of the wider language system, unlike the domain-general systems of attention and memory.

Defining the extent to which syntax is domain-specific has recently become important in the context of investigating the evolutionary precursors to human language. This research is based on the premise that the core aspect of human language processing is the construction of hierarchical syntactic structures, and tests this hypothesis by comparing the sensitivity of humans and macaques to different kinds of sequence learning — ranging from simple adjacency relationships which are not expected to show species differences — to hierarchical syntactic structures which are. The results of these studies support the claim that both humans and non-human primates are sensitive to simple adjacency relationships whereas only humans are able to construct hierarchical syntactic structures [32]. This kind of distinction enables us to further refine the core, domain-specific aspects of human language as distinct from domain-general aspects which are shared with other species.

The phylogeny of the domain-specific aspects of human language remain unknown, although there are a range of hypotheses about their genesis, from the idea that they arose through a genetic mutation [33] to the neuroconstructivist view [34] that evolution may be driven primarily by increased plasticity for learning, such that domain-specific systems emerge over developmental time from an interaction between domain-general learning mechanisms and exposure to human language.

## The relationship between the domain-specific language system and domain-general systems

The broad domain-general systems such as those subserving attention and memory are clearly not restricted to language processing, but may be called upon within certain situations/contexts. For instance, the frontoparietal control network (sometimes termed the multiple demand network [16, 35]), salience network [36], and default mode or core network [26, 27, 37] are thought to be responsible for attentional control, alerting/bottom-up attentional capture, and memory/imagination/introspection, respectively. Occasionally, these networks are implicated in fMRI studies of language comprehension, for instance, when language processing is difficult due to temporary syntactic ambiguities ([6]; cf. [12^•^]) or involves the processing of longer story narratives [38]. However, to conclude that these systems are somehow assisting with language-specific processes (or that ‘executive control and language appear to be causally linked’ [4], p. 4) is likely to be incorrect.

First, we know that these domain-general systems cannot compensate for syntactic processes carried out by the left-lateralized frontotemporal syntax system since they do not appear to be recruited when the left hemisphere frontotemporal syntax system is impaired following brain damage [39]. In a study involving brain-damaged patients with left hemisphere damage, syntactic performance correlated with fMRI activity and grey matter integrity, but no regions outside the left hemisphere frontotemporal syntax system were recruited during syntactic processing [3^•^]. Moreover, it is not just the integrity of these regions themselves that are critical for syntactic processing, but also the functional connectivity between them [40] and the integrity of the dorsal and ventral white matter tracts that connect them [41]. Second, damage to domain-general networks does not lead to aphasia, though it may lead to language impairments which are limited to situations which tax these domain-general systems and the processes they subserve [42, 43].

Recently, it has been suggested that the hippocampus contributes to language processing in that patients with damage to the hippocampus show certain language impairments (e.g. problems with referential processing [44]) and there are specific sub-regions of the hippocampus which are preferentially activated by language [38]. These language-selective sub-regions were defined using the same localizer approach described above as being selectively engaged by language (e.g. [28]). However, these hippocampal regions were only weakly correlated (*r*'s = .05–.15) with fMRI activity in the cortical language network defined by the localizer. Further, this finding is challenged by a recent analysis of the fMRI movie-watching data mentioned above [25•, 45] which showed a differential effect of ageing on the hippocampus and language network. While cross-subject synchrony (a commonly used marker of intact processing of naturalistic stimuli [46, 47]) declined dramatically with age in the hippocampus (as well as the frontoparietal network and medial PFC), synchrony of the language network remained intact with age (based on Bayes Factors; [45]). If the hippocampus contributes to language processing, as argued by Blank and colleagues, then one would expect age to affect these two systems in a similar way. It may be that the hippocampus covaries with the language system in the study by Blank and colleagues because *language* is what is being encoded by the hippocampus.

## Concluding comments

In this brief overview, we have suggested that it is necessary to take a more nuanced approach to differentiating domain-general and domain-specific components involved in language. While syntax seems to meet the criteria for domain-specificity in that it is fast, obligatory, and underpinned by a specialized neural system (see [48]), there are other key components in the wider language system (e.g. semantics and pragmatics) which are domain-general in that they are also involved in a number of cognitive functions which do not involve language. In addition, processing language under difficult conditions such as noisy environments [49], or within the context of a task [12•, 13], can spontaneously recruit broad domain-general networks. However, these differ from the domain-general networks of semantics and pragmatics. While the latter are key components of the broader language system and likely interact with each other and syntax during the processing of natural language [50, 51], domain-general systems such as attention and memory are not required for language comprehension and do not penetrate the domain-specific syntax system.

## Conflict of interest statement

Nothing declared.

## References and recommended reading

Papers of particular interest, published within the period of review, have been highlighted as:

• of special interest

## Footnotes

3 Some studies have shown substantial overlap in activation patterns associated with reading and listening to sentences [30•, 52], but there are also some differences between these conditions (see Figure 8 in [30^•^] and Figures 1 and S1 in [52]). These differences may be critical. Moreover, activation patterns associated with passive listening/reading [52] appear to be less extensive than those seen during task-based language processing (i.e. when a memory probe is used; e.g. Experiment 2 in [30^•^]), and it is this *task-based* version that is most commonly used by Fedorenko and colleagues (e.g. [4, 9, 28, 29]).
